# A Method of Differentiating Nerve Tissue with Mercuric Chloride

**Published:** 1891-10

**Authors:** 


### A Method of Differentiating Nerve Tissue with Mercuric Chloride.

Prof. Camillo Golgi (*Riforma Medica*, June 24, 1891), describes a new method of coloring nerve elements which may be of some value in pathological inquiries. The pieces of tissue are first hardened in Mueller's fluid and then in 3 per cent. potassum bichromate solution, in which they are left for several months. They are next soaked in a solution of corrosive sublimate, $1\frac{1}{2}$ to 1 per cent. It will then be found, on section, that the nervous elements have a glistening white appearance from impregnation with mercury, and are beautifully differentiated from the surrounding tissues. The author found, however, that a much better result was obtained if the sections were then immersed in a weak solution of sulphide, as the white appearance then disappeared and the nervous elements appeared of a fine black color, which showed up well in contrast with the surrounding tissues. The exact process adopted is as follows:

1. The sections having been cut in celliodin, are washed in distilled water.

2. They are then immersed for one or two minutes in a solution of sodium hyposulphite, preferably that used for "fixing" photographs on aristotype paper. Blackening can be then followed with the naked eye.

3. The sections are then very thoroughly washed in distilled water.

4. They may, if desired, be counter-stained with acid carmine.

5. After dehydration with absolute alcohol and clearing with oil of cloves they may be mounted in the ordinary manner.

Sections thus treated are very permanent; even after prolonged keeping they do not show pulvirulent deposits, and the nervous elements are shown in their finest ramifications.—*British Medical Journal.*

